# The Role of Exogenous Gibberellic Acid and Methyl Jasmonate against White-Backed Planthopper (*Sogatella furcifera*) Stress in Rice (*Oryza sativa* L.)

**DOI:** 10.3390/ijms232314737

**Published:** 2022-11-25

**Authors:** Saleem Asif, Yoon-Hee Jang, Eun-Gyeong Kim, Rahmatullah Jan, Sajjad Asaf, Muhammad Aaqil Khan, Muhammad Farooq, Lubna, Nari Kim, In-Jung Lee, Kyung-Min Kim

**Affiliations:** 1 Department of Applied Biosciences, Graduate School, Kyungpook National University, Daegu 41566, Republic of Korea; 2 Coastal Agriculture Research Institute, Kyungpook National University, Daegu 41566, Republic of Korea; 3 Natural and Medical Science Research Center, University of Nizwa, Nizwa 616, Oman; 4 Department of Botany, Garden Campus, Abdul Wali Khan University, Mardan 23200, Pakistan

**Keywords:** exogenous hormones, white-backed planthoppers, gibberellic acid and methyl jasmonate, antioxidant, biotic stress

## Abstract

Rice (*Oryza sativa* L.) is one of the essential staple foods for more than half of the world’s population, and its production is affected by different environmental abiotic and biotic stress conditions. The white-backed planthopper (WBPH, *Sogatella furcifera*) causes significant damage to rice plants, leading to substantial economic losses due to reduced production. In this experiment, we applied exogenous hormones (gibberellic acid and methyl jasmonate) to WBPH-infested rice plants and examined the relative expression of related genes, antioxidant accumulation, the recovery rate of affected plants, endogenous hormones, the accumulation of H_2_O_2_, and the rate of cell death using DAB and trypan staining, respectively. The expression of the transcriptional regulator *(OsGAI*) and gibberellic-acid-mediated signaling regulator (*OsGID2*) was upregulated significantly in GA 50 µM + WBPH after 36 h. *OsGAI* was upregulated in the control, GA 50 µM + WBPH, GA 100 µM + WBPH, and MeJA 100 µM + WBPH. However, after 48 h, the *OsGID2* was significantly highly expressed in all groups of plants. The glutathione (GSH) values were significantly enhanced by GA 100 µM and MeJA 50 µM treatment. Unlike glutathione (GSH), the catalase (CAT) and peroxidase (POD) values were significantly reduced in control + WBPH plants. However, a slight increase in CAT and POD values was observed in GA 50 + WBPH plants and a reduction in the POD value was observed in GA 100 µM + WBPH and MeJA 50 µM + WBPH plants. GA highly recovered the WBPH-affected rice plants, while no recovery was seen in MeJA-treated plants. MeJA was highly accumulated in control + WBPH, MeJA 50 µM + WBPH, and GA 100 µM + WBPH plants. The H_2_O_2_ accumulation was highly decreased in GA-treated plants, while extensive cell death was observed in MeJA-treated plants compared with GA-treated plants. From this study, we can conclude that the exogenous application of GA can overcome the effects of the WBPH and enhance resistance in rice.

## 1. Introduction

Rice (*Oryza sativa* L.) is a major staple food for more than half of the world’s population, with huge importance in Asia. It is cultivated in more than 100 countries and is a basic source of income and employment for rural people [1]. Rice grows in warm and humid conditions, which are very compatible with pest proliferation. The white-backed planthopper (WBPH, *Sogatella furcifera* (Horvath)), is one of the serious and emerging pathogenic pests in Asian countries, especially in China and Korea. The WBPH was reported in India for the first time in 1966 and it spread to China, Korea, and Japan within a very short timeframe. It causes significant destruction to rice plants at the seedling stage and causes a severe loss of yield. A report has shown that it causes a 10–20% yield loss in China each year [2]. Due to the short lifespan, the WBPH population spreads rapidly in the invaded locality. Due to a lack of controlling measures implemented during the initial stage, they spread within the invaded region quickly, and are then difficult to control [1].

The WBPH penetrates the plant tissue an’ removes cell sap through sucking, transferring viruses such as grassy stunt and rugged stunt, causing wilting or a loss of green color [3]. Before 2001, the WBPH was not considered to be a viral vector, but rather a sap-sucking insect that caused nutrient deficiency; in 2001, a new virus was reported, namely the southern rice black-streaked dwarf virus, which is transmitted by the WBPH, which caused a great loss of hybrid rice in China and Vietnam [3,4]. The WBPH mostly feeds on the stem of the rice plant and damages the rice plants by sap-sucking and inserting their feeding sheath into the xylem and phloem [5]. Previous studies have reported that phloem sap-sucking pests use the host plant sugar content as a source of energy [6].

Many pathogens frequently attack plants, and to face such adverse situations, the host plants activate their defense mechanisms, immune system, and defense-related gene expression to enhance resistance to the pathogen. The host plants also produce different types of hormones, which result in defense responses to different stresses. In response to biotic stress, plants mainly produce salicylic acid (SA), jasmonate (JA), ethylene (ET), and gibberellic acid (GA) to overcome the effects of biotic stress [7,8]. Gibberellic acid (GA) and methyl jasmonate (MeJA) are plant-growth-promoting hormones that play a regulatory role in plant growth and development; besides growth, both play a key role in stress tolerance. Gibberellic acid is biosynthesized from geranylgeranyl diphosphate, which controls seed germination, stem elongation, leaf expansion, trichome development, and seed and flower development [9,10]. Genetic studies have revealed several GA signaling components. Some of them include the GA-insensitive *dwarf 2* (*OsGID2*). A class of repressors called DELLA proteins belonging to the GRAS family of transcription factors are also negative regulators of GA signaling. A single DELLA protein is present in rice known as SLENDER RICE1 (SLR1) and its function is to repress every aspect of the GA response. Under stress conditions, DELLAs help in the defense response [8].

Jasmonate plays a key role in the defense against insects and pathogens, and in reproductive development [11]. *OsPOX* and *OsRBBI3-3* are JA-related genes involved in the defense response, induced by two potent protein phosphatase 2A (PP2A) molecules, playing a key role in kinase signaling cascades in their expression. Plants continuously sense the level of ROS and alter their gene expression to respond to different environmental stresses [12]. Rice also responds to the WBPH through the activation of oxidative and phenylpropanoid pathway enzymes. Antioxidants, such as peroxidase (POD), glutathione (GSH), and catalase (CAT), also increase pest resistance by modifying the cell wall structure [13]. Therefore, we hypothesized that GA and MeJA can significantly reduce WBPH damage in rice plants. In this study, we aimed to demonstrate that the exogenous application of GA and MeJA inhibits the oxidative stress induced by pests via regulation of antioxidant defense systems and regulation of endogenous hormonal crosstalk.

## 2. Results

### 2.1. Measurement and Analysis of Agronomic Traits

**Figure 1:**
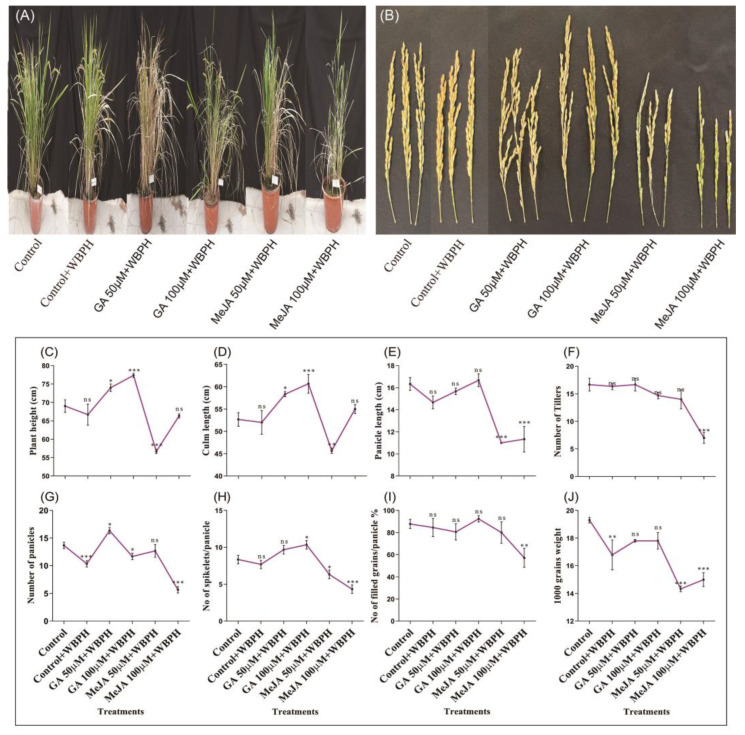
Agronomic traits of the plants at the mature stage receiving different treatments. (**A**) Pictorial representation of plant height. (**B**) Pictorial representation of panicle length. (**C**) Plant height. (**D**) Culm length. (**E**) Panicle length. (**F**) The number of tillers. (**G**) The number of panicles. (**H**) The number of spikelets per panicle. (**I**) The filed grain percentage. (**J**) 1000-grain weight. Each result is the mean of three replicates. The error bars represent the standard error of the mean data (*n* = 3); ns represents not significant, and asterisks indicate a significant difference (* *p* < 0.05, ** *p* < 0.01, *** *p* < 0.001), according to the analysis using two-way ANOVA and the Bonferroni post hoc test. The experiments were repeated three times.

In this study, different agronomic traits were analyzed under WBPH stress treated with GA and MeJA hormones. We observed a 7% and 12% increase, and a −18% decrease in plant height in GA 50 µM + WBPH, GA 100 µM + WBPH, and MeJA 50 µM + WBPH plants, respectively (Figure 1A,C). Culm length was increased by 11% and 15% in GA 50 µM + WBPH and GA 100 µM + WBPH plants, respectively, while a −13% decrease was observed in MeJA 50 µM + WBPH plants (Figure 1D). Panicle length was reduced by −33% and −31% in MeJA 50 µM + WBPH and MeJA 100 µM + WBPH plants, respectively (Figure 1B,E). The number of tillers was reduced by 58% in MeJA 100 µM + WBPH plants, while no significant change was seen in the other plants (Figure 1F). The number of panicles was increased by 20% in GA 50 µM + WBPH plants, while a −24%, −15%, and −59% reduction was seen in the control + WBPH, GA 100 µM + WBPH, and MeJA 100 µM + WBPH plants, respectively (Figure 1G). The number of spikelets increased by 24% in GA 100 µM + WBPH plants, while a −24% and −48% decrease was observed in MeJA 50 µM + WBPH and MeJA 100 µM + WBPH plants, respectively (Figure 1H). No significant increase was seen in the percentage of filled grain per panicle, while a −35% decrease was seen only in MeJA 100 µM + WBPH plants (Figure 1I). A significant decrease in the 1000-grain weight of −13%, −26%, and −22.27% was seen in control + WBPH, MeJA 50 µM + WBPH, and MeJA 100 µM + WBPH plants, respectively, while no reduction was seen in GA-treated plants (Figure 1J).

### 2.2. Relative Gene Expression

**Figure 2:**
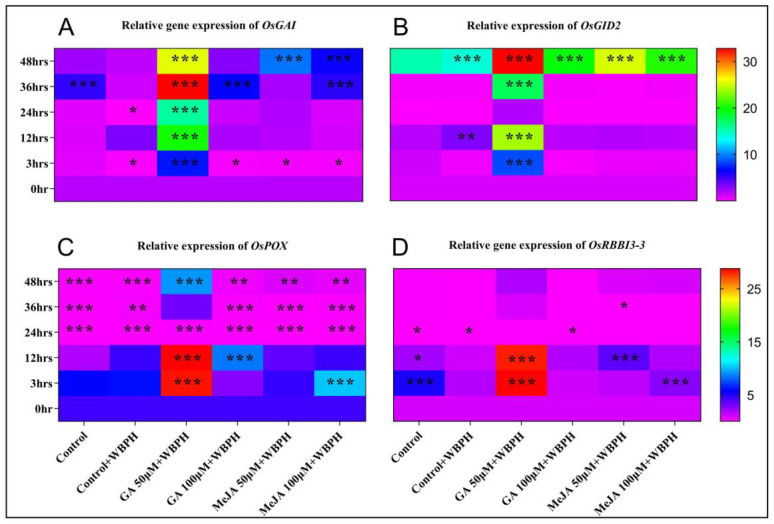
Relative gene expression of related genes in the plants in different treatment groups. (**A**) *OsGAI*. (**B**) *OsGID2*. (**C**) *OsPOX*. (**D**) *OsRBBI3-3*. The relative expression of each gene was measured after 0, 6, 12, 24, 36, and 48 h of WBPH infestation and hormone treatment. The actin gene was used as the reference gene. Each result is the mean of three replicates. Error bars represent the standard error of the mean data (*n* = 3). The error bars with no asterisks indicate non-significant differences, and asterisks indicate a significant difference (* *p* < 0.05, ** *p* < 0.01, *** *p* < 0.001), according to analysis using two-way ANOVA and the Bonferroni post hoc test. The experiments were repeated three times.

We evaluated the expression of *OsGAI*, *OsGID2*, *OsPOX*, and *OsRBB13-3* against WBPH stress. These gene expressions were quantified after 0, 3, 12, 24, 36, and 48 h of WBPH infestation and hormone treatment (Figure 2). We found that *OsGAI* was thoroughly upregulated in plants treated with GA 50 µM + WBPH after the infestation with WBPHs and GA treatment (Figure 2A). After 3 h of hormone treatment, the *OsGAI* gene was suppressed in all other groups of plants except those treated with GA 50 µM + WBPH, where it increased by 277% (Figure 2A). We found that *OsGAI* expression increased 1633% and 1251% in GA 50 µM + WBPH, 248% and 67% in GA 100 µM + WBPH, and 195% and 236% in MeJA 100 µM + WBPH plants after 36 and 48 h, respectively (Figure 2A). Similarly, the *OsGID2* gene was thoroughly expressed in GA 50 µM + WBPH plants after WBPH infestation and hormone treatment. *OsGID2* expression increased by 748% and 2291% in GA 50 µM + WBPH plants after 3 and 12 h, respectively. However, after 24 h, it showed no expression; although, expression increased by 1645% after 36 h, and 3177% after 48 h. No significant expression was seen in the other groups of plants before 48 h, but the gene was significantly expressed in all groups of plants after 48 h (Figure 2B). We also found that the *OsPOX* gene was highly expressed, at 560% and 570%, in plants treated with GA 50 µM after 3 and 12 h, respectively. The *OsPOX* expression increased by 139% in MeJA 100 µM plants after 3 h and 97% in GA 100 µM plants after 12 h. Inversely, after 24, 36, and 48 h, the gene was significantly downregulated in all groups of plants except GA 50 µM plants, in which the gene was significantly expressed again after 48 h at 113% (Figure 2C). The *OsRBBI3-3* gene was expressed in plants treated with GA 50 µM after 3 and 12 h, at 2778% and 2716%, respectively. Significant upregulation was seen in the control plants after 3 and 12 h (419% and 110%, respectively), in MeJA 100 µM plants after 3 h (167%), and in MeJA 50 µM plants after 12 h (267%). At the same time, downregulation was seen in some groups of plants after 24 h (Figure 2D).

### 2.3. Histochemical Analysis and Antioxidant Accumulation in Response to WBPH Stress and Applying Hormones

**Figure 3:**
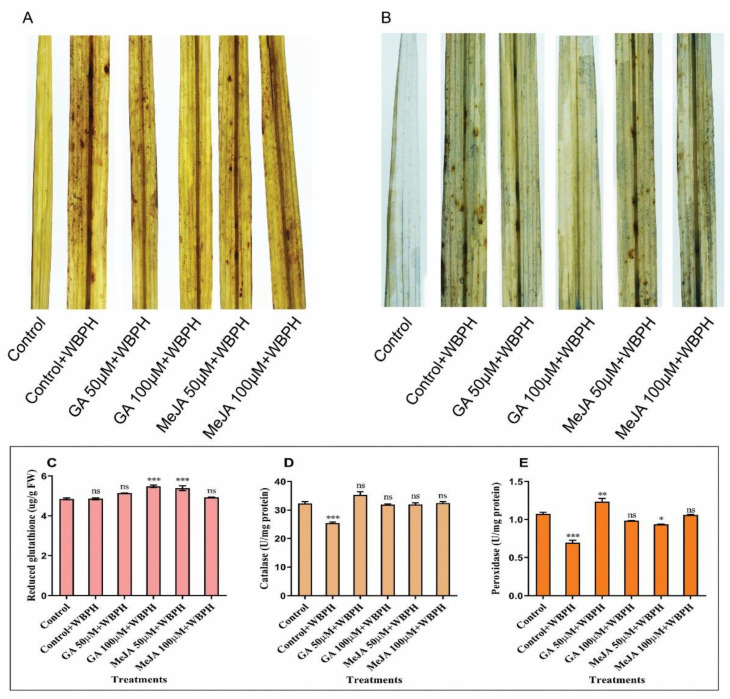
Effects of GA and MeJA applications on white-backed planthopper (WBPH) stress through the induction of hypersensitive responses and antioxidant activities. (**A**) DAB staining for H_2_O_2_ accumulation. (**B**) Trypan blue staining for detection of dead cells. (**C**) GSH, (**D**) CAT, and (**E**) POD accumulation in response to GA and MeJA applications toward WBPH infestation. Each result is the mean of three replicates. Error bars represent the standard error of the mean data (*n* = 3), ns represents not significant, and asterisks denote a significant difference (* *p* < 0.05, ** *p* < 0.01, *** *p* < 0.001), according to the analysis using two-way ANOVA and the Bonferroni post hoc test. The experiments were repeated three times.

The hypersensitive response (HR) of GA and MeJA in response to WBPH stress was evaluated by using diaminobenzidine (DAB) staining and trypan blue staining, respectively (Figure 3). The H_2_O_2_ accumulation was visualized in MeJA-treated plants and was similar to that of the control + WBPH plants; however, GA 50 µM + WBPH plants had low H_2_O_2_ accumulation, as compared with the control + WBPH plants, while GA 100 µM + WBPH plants showed no H_2_O_2_ accumulation. These quantitative images showed less accumulation of H_2_O_2_ in GA-treated plants compared with other plants. Leaf samples were collected from all groups of plants in the mature stage and compared with control plants (Figure 3A). Cell death was detected in both MeJA- and GA-treated plants. Our results showed that GA 50 µM + WBPH plants had a low amount of cell death; however, GA 100 µM + WBPH plants showed no cell death compared with control plants. This extensive cell death in MeJA-treated plants shows that MeJA has no defensive response to WBPH infestation, while GA shows that it reduced the amount of cell death by reducing the effect of WBPH infestation (Figure 3B).

Furthermore, antioxidant analysis showed that GSH values were significantly increased 6% and 13% by GA treatment in GA 50 µM + WBPH and GA 100 µM + WBPH, respectively (Figure 3C). Unlike GSH, the CAT value was significantly reduced by 21% in control + WBPH plants compared with the control. However, a slight increase in the CAT value was observed in GA 50 µM + WBPH plants (Figure 3D). Similarly, the POD value was reduced −35% and −13% in control + WBPH and MeJA 50 µM + WBPH plants, respectively. In contrast, a 15% increase was found in GA 50 µM + WBPH plants, as shown in Figure 3E.

### 2.4. Measurement of Chlorophyll Content, RWC, and Electrolyte Leakage

**Figure 4:**
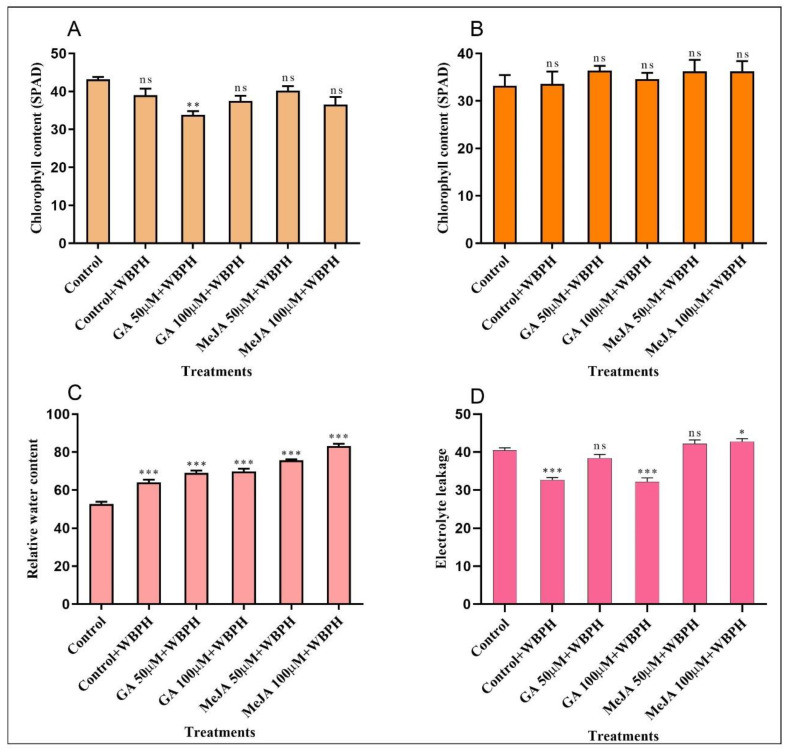
Measurement of chlorophyll content, RWC, and electrolyte leakage. (**A**) Chlorophyll contents after 10 days of WBPH inoculation and hormone treatment. (**B**) Chlorophyll contents after 30 days of WBPH inoculation and hormone treatment. (**C**) Relative water content. (**D**) Electrolyte leakage. Each result is the mean of three replicates. Error bars represent the standard error of the mean data (*n* = 3), ns represents not significant, and asterisks denote a significant difference (* *p* < 0.05, ** *p* < 0.01, *** *p* < 0.001), according to the analysis using two-way ANOVA and the Bonferroni post hoc test. The experiments were repeated three times.

Chlorophyll contents were measured two times, one after 10 days of WBPH infestation and the other after 30 days. The results obtained after 10 days showed a decrease in the chlorophyll contents in all groups compared with the control (Figure 4A). While, after 30 days, a significant decline was observed in chlorophyll contents compared with the results after 10 days. However, after 30 days, a significant change was observed in all groups of plants compared with the control plants, as shown in Figure 4B. We found that RWC was significantly increased by 21%, 31%, 32%, 43%, and 58% in control + WBPH, GA 50 µM + WBPH, GA 100 µM + WBPH, MeJA 50 µM + WBPH, and MeJA 100 µM + WBPH, respectively, as shown in Figure 4C. It is well known that an increase in ROS causes electrolyte leakage due to oxidative damage. To investigate the role of GA and MeJA treatment, we quantified the electrolyte leakage in the control and all the treated plants. In control + WBPH (−19%) and GA 100 µM + WBPH (−20%) plants, the electrolyte leakage was significantly decreased, while no significant change was observed in the other groups of plants as compared with the control plants, as shown in Figure 4D.

### 2.5. Recovery of Infected Plants after Hormone Treatment

**Figure 5:**
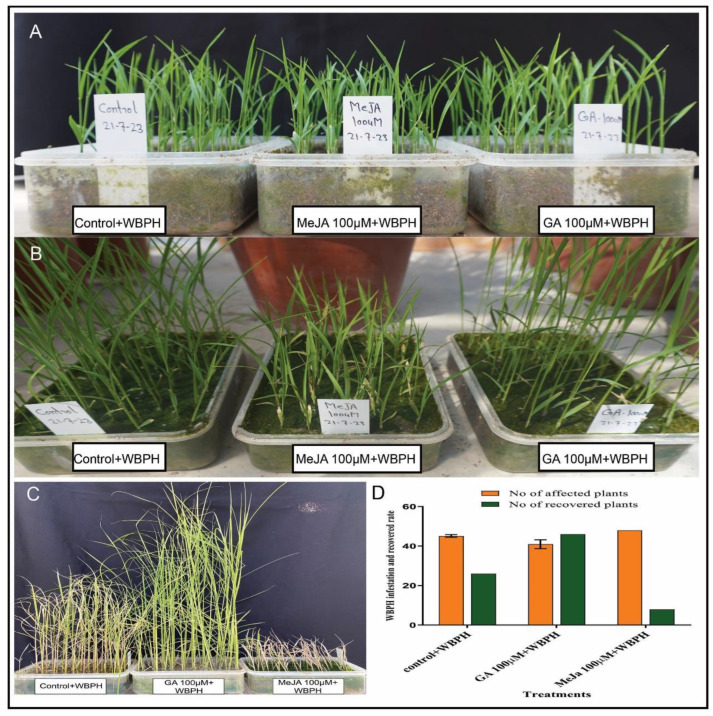
The recovery rate of WBPH-affected plants by exogenous GA and MeJA hormones. (**A**) The plants before WBPH infestation and hormone treatment. (**B**) After 7 days of WBPH stress and continuous treatment with 100 µM GA and 100 µM MeJA. (**C**) Recovery of plants after 7 days. (**D**) Graphical representation of affected and recovered plants after WBPH stress by applying exogenous hormones. Error bars represent the standard error of the mean data (*n* = 3).

Our results showed that, after 7 days of WBPH infestation, all plants were infected; after that, insects were removed and hormones were applied exogenously to check the recovery rate of plants. After hormonal treatment for 7 days, the GA-treated plants showed significant recovery; nearly all the plants were recovered and increased their height. However, the MeJA-treated plants showed no recovery and remained infected (Figure 5). The number of insects was also counted daily, and the control plants had a high number of insects, followed by GA-treated plants; however, the MeJA-treated plants had a lower number of insects on all 7 days, as shown in (Table 1).

### 2.6. Quantification of Endogenous GA and MeJA Hormones

**Figure 6:**
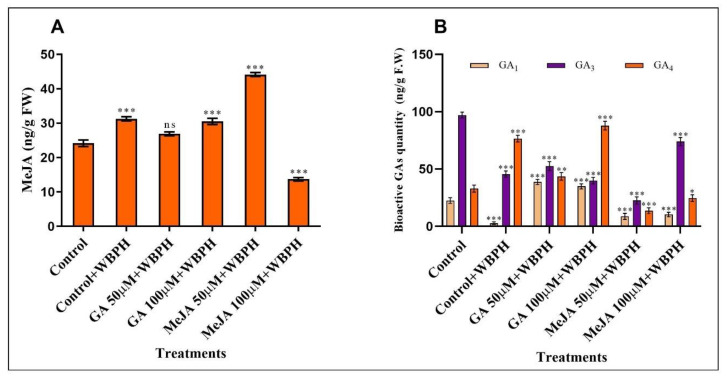
Quantification of endogenous hormones. (**A**) Quantification of endogenous MeJA. (**B**) Quantification of bioactive GAs (GA1, GA3, and GA4). Error bars represent the standard error of the mean data (*n* = 3); ns represents not significant, and asterisks denote a significant difference (* *p* < 0.05; ** *p* < 0.01; *** *p* < 0.001), according to the analysis using two-way ANOVA and the Bonferroni post hoc test. The experiments were repeated three times.

The endogenous quantification of GA and MeJA was analyzed. A significant increase in MeJA was found in control + WBPH, GA 100 µM + WBPH, and MeJA 50 µM + WBPH plants, while no significant change was seen in GA 50 µM + WBPH plants. However, a highly significant decrease was found in MeJA 100 µM + WBPH plants (Figure 6A). Endogenous bioactive GA was quantified in the control plants and all treated plants. GA1 was significantly reduced in control + WBPH, MeJA 50 µM + WBPH, and MeJA 100 µM + WBPH plants, while a significant increase was observed in GA-treated plants. GA3 was significantly reduced in all WBPH-infested plants compared with control plants; in contrast to other plants, the MeJA 100 µM + WBPH plants showed a lower reduction in GA3. Similarly, GA4 was significantly increased in control + WBPH, GA 50 µM + WBPH, and GA 100 µM + WBPH plants, while a high reduction was seen in MeJA-treated plants (Figure 6B).

## 3. Discussion

Plants have evolved a highly developed immune system in order to protect against invasive pathogens. The primary support structure for plant basal immunity is comprised of plant hormones such as salicylic acid (SA), jasmonate (JA), and ethylene (ET) [14,15]. Other plant hormones, including auxin, GA, ABA, brassinosteroids (BR), cytokinins (CK), and brassinosteroids (BR), that were previously believed to only be involved in abiotic stress or the regulation of plant growth and development, have also been ascribed a role in plant immunity [16,17]. Owing to its action throughout the plant life cycle, GA is thought to play a significant role in the development and regulation of plant growth. However, the use of GA in the study of plant disease physiology is an emerging field [18,19]. Previous reports revealed that GA biosynthesis and signaling genes were upregulated in giant cells induced by *M. graminicola* in rice [20]. Our results revealed that genes related to GA and methyl jasmonate biosynthesis were regulated after GA treatment. Previous studies have reported that the expression of the gibberellin-insensitive gene (*OsGAI)* was induced after 6 h of exogenous application of GA [21]. OsGAI was believed to be an early negative regulator that activates signaling pathways linked to defense and exhibits a strong resistance to BPH, since it was downregulated in response to female BPH [22].

In contrast, our data suggested that *OSGAI* was significantly upregulated by GA treatment and not downregulated by WBPH infestation. *OsGID2* is considered a positive regulator for GA signaling, encoding an F-box protein and SLR1 [23]. In our study, the *OsGID2* gene was continuously induced in GA 50 µM + WBPH plants after 3 and 12 h, whereas no significant expression was found in other treatments until 48 h. Similar results observed previously have shown that *OsGID2* was upregulated by the application of exogenous GA hormone [24]. On the other hand, *OsPOX* is a JA-related gene involved in the defense response induced by two potent protein phosphatase 2A (PP2A) molecules. It plays a pivotal role in kinase signaling cascades in their expression [25,26]. In our study, the *OsPOX* gene was significantly induced by GA and MeJA treatment in response to WBPH infestation. These results are in agreement with previous reports [27]. *OsBBI3-3*, a proteinase inhibitor belonging to the Bowman–Birk family, shows a response to stress and exogenous jasmonic acid treatment [26]. In our study, the *OsRBBI3-3* gene was significantly induced in control, GA-treated, and MeJA-treated plants after 3 h and 12 h. These results are similar to previous findings that have shown that applying exogenous MeJA to infected plants significantly induced *OsRBBI3-3* gene expression [27].

Rice consistently develops robust machinery to counteract the harmful effects of ROS and handle various stressors, including WBPH infestation [28]. As one of the physiological responses to herbivory [29], malondialdehyde (MDA) concentration often rises and it has been widely employed as a biomarker of the severity of cell membrane damage [30]. A variety of plant defense systems are activated in the early stages of a plant’s response to biotic stress by the plant’s fast and transient production of reactive oxygen species (ROS), especially H_2_O_2_ [31]. These antioxidant activities increase in response to pathogen infestation, which increases resistance to the pathogen [13,32]. Glutathione (GSH), catalase (CAT), and peroxidase (POD) are antioxidant enzymes crucial for maintaining a balance of ROS, and they have been found to be more active in GA-treated plants than in non-treated infected plants [33]. Similar results were observed in another study showing improved growth of stressed pea plants due to regulation of the antioxidant activities by exogenous application of GA [34]. Our experiment found that GSH values were significantly induced by GA and MeJA treatment. The CAT value was significantly reduced in control + WBPH plants; however, no significant change was seen in other plants. Similarly, the POD value was reduced in control + WBPH plants due to WBPH stress; however, a slight increase was observed in GA 50 µM + WBPH plants. Our results agreed with the previous reports which suggest induction of antioxidant activity against WBPH infestation in rice plants by application of exogenous hormones (Figure 3).

H_2_O_2_ and other organic peroxides are reduced by GPx catalases using glutathione as a reducing agent. The WBPH generates ROS in rice tissues, leading to cell death [1]. Histochemical staining with DAB and trypan indicates that H_2_O_2_ accumulation was reduced in GA-treated plants and a lower amount of cell death was visualized in GA-treated plants. Previous studies suggested that high H_2_O_2_ accumulation and cell death were seen in WBPH-infested plants [7]. Our study suggests that giving exogenous GA treatment to WBPH-infested plants can reduce these effects, which is in line with previous reports that applying exogenous GA can reduce the intracellular ROS under stress conditions [35].

The WBPH infestation greatly reduced the yield of rice plants by up to 40% by inserting viral material into the plant body, directly feeding on phloem sap, causing wilting or a loss of green color, and in severe cases causing plant death [36]. GA is a growth-promoting hormone that can improve the yield of plants [37]. Previous studies suggest that the exogenous application of GA can increase plant height and biomass; however, no significant increase was seen in other agronomic traits [37]. Methyl jasmonate is an important plant hormone involved in many plant developmental processes and it responds to insect wounding and attacks from different pathogens [38]. However, previous studies also suggest that applying methyl jasmonate exogenously in response to biotic and abiotic stress is still controversial [39]. We found that GA enhanced growth parameters such as plant height, culm length, and panicle length, while MeJA reduced these parameters. The results suggested that GA application significantly promotes agronomic traits under WBPH stress, compared with MeJA application.

It has been reported that the exogenous application of GA regulates the endogenous levels of GA1 and GA4, and other hormones in response to stress [40]. Studies have also revealed that the level of endogenous JA increases in response to pest inoculation [41], but some studies have suggested that exogenous MeJA did not increase the level of endogenous MeJA in rice [42]. In our study, we found that the level of MeJA was significantly increased in control + WBPH plants as well as in GA 100 µM + WBPH and MeJA 50 µM + WBPH plants. This increase may be due to pest inoculation; however, in MeJA 100 µM + WBPH plants, the level was significantly reduced. In our study, the exogenous application of MeJA and WBPH stress both inhibited the formation of GA1, but the exogenous application of GA enhanced it. However, WBPH infestation significantly reduced GA3. GA4 levels decreased in MeJA-treated plants, while they increased significantly in WBPH-infested and GA-treated plants. Similar results have been reported previously by [43] who showed that the application of exogenous GA3 promoted endogenous GA3 production. Feedback regulation of GA biosynthesis genes, such as PsCPS, PsGA2ox, and PsGA3ox, as well as GA signaling genes, such as PsGID1b/c, PsGAI, and PsGID2, regulate endogenous levels of GAs.

## 4. Materials and Methods

### 4.1. Plant Selection and Growth Conditions

*Oryza sativa* L., cultivar ‘Ilmi’ was selected for the current experiment; the seeds were provided by the Plant Molecular Breeding Laboratory, Kyungpook National University, South Korea. The seeds were treated with 500 µL of fungicides and were soaked in water for three days at 34 °C, in the incubator in the dark. The water was changed daily. After three days of soaking, the pre-germinated seeds were transferred to soil and kept in the dark for three days until successful growth was observed, as published previously [7], and the seedlings were kept in the greenhouse for further experimentation.

### 4.2. Experimental Design

Six groups of Ilmi plants were selected to evaluate the exogenous effects of GA and MeJA treatment against the WBPH on rice plants. We grew plants in pots, and then 21- day-old plants were transferred to 21 × 15 cm pots according to the experimental design, with three replicates in each group. At the same time, plants were infested with WBPHs which were starved two hours before infestation and were treated with hormones as shown in Table 2. The plants were kept in the insectarium and were inoculated with 50 insects per plant. The WBPH population was obtained from the Rural Development Administration Centre in Jeongu, Korea. The WBPH were reared with special care in a transparent glass cage, measuring 50 cm × 50 cm × 40 cm in terms of length, width, and height, respectively.

### 4.3. Measurement and Analysis of Agronomic Traits

Agronomics traits such as panicle length, culm length, culm + panicle length, panicle number, tiller number, leaf width, number of spikelets per panicle, filled grain percentage, and 1000-grain weight were measured.

### 4.4. RNA Isolation and qRT-PCR

The leaf samples were collected for total RNA extraction from the plants of all six groups in triplicate, after 0, 6, 12, 24, 36, and 48 h of WBPH infestation and hormone treatment, to check the relative expression of the selected genes. For first-strand cDNA synthesis, the qPCRBIO cDNA Synthesis Kit and 500 ng of total RNA were used. For quantitative RT-PCR, we used the StepOnePlus Real-Time PCR System, Life Technologies Holdings Pte Ltd. (Singapore), BioFACT™ 2X Real-Time PCR Master Mix (including SYBR^®^ Green I), (www.bio-ft.com; South Korea), and primers specific to the selected genes (Table 3). *OsActin1* (accession no. X16280.1) was used as an internal reference gene for normalization.

### 4.5. Histochemical Analysis

The hypersensitive response (HR) of GA- and MeJA-treated plants toward WBPH infestation was compared with control plants according to [44]. Leaves from all groups at the same tillering stage were cut off and placed in glass tubes containing trypan blue staining solution and boiled for 10 min; this was followed by keeping them in the dark for 12 h. The leaves were then kept in 25 mg/mL chloral hydrate for 24 h to remove the color. Blue spots on the leaves were recorded and photographed. Following the method of [45], the accumulation of H_2_O_2_ was measured using DAB staining.

### 4.6. Estimation of Antioxidant Activities

The activity of antioxidant enzymes, such as glutathione (GSH), catalase (CAT), and peroxidase (POD), was measured. GSH was measured according to the method described by [46]. Briefly, 200 mg samples were powdered and homogenized in 3 mL of 5% trichloroacetic acid (TAC), followed by centrifugation at 10,000× *g* for 15 min. The (0.1 mL) supernatant was transferred to (3 mL) 150mM monosodium phosphate buffer and (0.5 mL) Ellman’s reagent, followed by incubation at 30 ± 2 °C for 5 min. Then, absorbance was measured spectrophotometrically at 412 nm. CAT activity was measured following the method described by [47], and the absorbance was measured spectrophotometrically at 240 nm. The POD was measured followed the method used by [48]. Briefly, 400 mg samples were powdered using a chilled mortar and pestle. A 0.1 M potassium phosphate buffer (pH 6.8) was added to the samples and centrifuged at 4 °C for 15 min at 5000 rpm in a refrigerated centrifuge. A reaction mixture containing 0.1 M potassium phosphate buffer (pH 6.8), 50 µL pyrogallol (50 µM), and 50 µL H_2_O_2_ (50 µM) was mixed with 100 µL of the sample crude extract, and the reaction mixture was incubated at 25 °C for 5 min. After incubation, 5% H2SO4 (*v*/*v*) was added to stop the enzymatic reaction. The resulting absorbance was measured at 420 nm. One unit of POD was directly measured by an increase of 0.1 units of absorbance.

### 4.7. Measurement of Chlorophyll Content

The chlorophyll content was measured at 10 and 30 days after inoculation with WBPHs and treatments. A portable chlorophyll meter (SPAD 502, Konica Minolta, Japan) was used to measure the chlorophyll content. Five leaves were measured for each group, and each leaf was measured at three points: leaf tip, middle leaf, and leaf base. The average value was taken as the SPAD value of the leaf.

### 4.8. Relative Water Content and Electrolyte Leakage

The leaf relative water content was measured in fully mature leaves of three plants per group using the method of [49]. The leaves were taken, and their fresh weight (FW) was recorded immediately. The leaf samples were incubated on distilled water in Petri dishes for 3 h to regain their turgidity, and the turgid weight (TW) was recorded. The samples were then dried at 70 °C for 48 h and the dry weight (DW) was measured. The relative water content (RWC) was measured using the following formula:

$$\mathrm{RWC}=\frac{\mathrm{Fresh}\;\mathrm{weight}-\mathrm{Dry}\;\mathrm{weight}}{\mathrm{Turgid}\;\mathrm{weight}-\mathrm{Dry}\;\mathrm{weight}}\times 100$$

### 4.9. Electrolyte Leakage

Electrolyte leakage was determined following the method of [50] using a Huriba B-173 Twin Cond electrical conductivity meter (Minami-Ku, Kyoto, Japan). The mature leaves were cut into 5 mm segments and immersed in 25 mL distilled water in 50 mL centrifuge tubes. The samples were incubated for 24 h at 25 °C and the initial conductivity (EC1) of the solutions was measured. The samples and solutions were then boiled for 30 min in water and were cooled to room temperature, and electrical conductivity (EC2) was measured. EL was calculated using the following formula:

$$\mathrm{EL}=\frac{\mathrm{EC}1}{\mathrm{EC}2}\times 100$$

### 4.10. Recovery of Infected Plants

We performed another experiment to determine the recovery of plants after the infestation with WBPHs. We selected the first group as the control plants, the second group of plants was treated with GA, and the third group of plants was treated with MeJA. A total of 50 two-week-old plants from each group were infested with WBPHs for one week. After one week, the number of WBPHs were counted and then the plants were treated with their relative hormones to evaluate the infected plants’ recovery rate.

### 4.11. Quantification of Endogenous GA and MeJA Hormones

To quantify GA and MeJA in all groups of plants, in response to WBPH stress, mature leaves of plants from all groups were collected in liquid nitrogen and were stored at −80 °C. For quantification, frozen leaves were crushed into a fine powder in liquid nitrogen using a chilled mortar and pestle. For MeJA analysis, 200 mg of freeze-dried leaves were mixed with acetone and 50 mM citric acid (70:30, *v*/*v*), following the method of [51]. For GA1, GA3, and GA4 extraction and quantification, the well-established protocol proposed by [52] was used. Extracted GA1, GA3, and GA4 underwent reverse-phase C18-HPLC and were chromatographed on a 3.9 × 300 m Bondpak, C18 column (Waters Corp., Milford, MA, USA) followed by elution at 105 mL/min with the following gradient: 0 to 5 min, isocratic 28% MeOH in 1% aqueous acetic acid; 5 to 35 min, linear gradient from 28 to 86% MeOH; 35 to 36 min, 86 to 100% MeOH; and 36 to 40 min, isocratic 100% MeOH. Through selected ion monitoring (SIM), the fractions were then subjected to a gas chromatograph/mass spectrometer (GC/MS). For GA1, GA3, and GA4 quantification, 1µL of each sample was injected in a 30 m × 0.25 mm (i.d.), 0.25 μm film thickness DB-1 capillary column (J & W Scientific Co., Folsom, CA, USA). The oven temperature for GC was set as: 1 min hold at 60 °C, then rising to 200 °C at 15 °C min^−1^, and finally reaching 285 °C at 5 °C min^−1^. Helium was used as a carrier gas, which maintained 30 kPa of head pressure. The GA1, GA3, and GA4 were calculated from the peak ratios of 508/506 and 286/284 *m/z*, respectively. All of the analyses were repeated three times.

### 4.12. Statistical Analysis

All the experiments were performed in triplicate, and the data from each replicate were pooled. Data were analyzed using two-way ANOVA, followed by the Bonferroni post hoc test (* denotes *p* < 0.05, ** denotes *p* < 0.01, *** denotes *p* < 0.001). A completely randomized design was used to compare the mean values of different treatments. Data are graphically presented, and statistical analyses were performed using the GraphPad Prism software (version 5.01, GraphPad, San Diego, CA, USA).

## 5. Conclusions and Future Perspective

In this study, we observed that the exogenous application of GA enhanced the pest tolerance of rice by reducing electrolyte leakage via activating antioxidant enzyme activities, upregulating GA-related gene expression, and increasing the bioactive GA level in plants. In addition, GA also enhanced the agronomic traits and played a key role in the recovery of affected plants. We also found that GA application reduced H_2_O_2_ accumulation and cell death. Therefore, we conclude that GA application can reduce the effects of the WBPH on rice. The finding provides a novel insight into the crosstalk of different hormones. To understand this interaction, further investigation is required to determine many other aspects of the role of GA in biotic stress.

## Figures and Tables

**Table 1:** The number of insect counts per day in different treatment groups.

Days	Control	100 µM GA	100 µM MeJA
1	135	84	45
2	135	48	19
3	140	20	27
4	125	21	30
5	125	22	30
6	105	25	24
7	95	26	23

**Table 2:** The design of different experimental groups.

No	Name	Stress	Treatment
1	Control	No	Only water
2	Control + WBPH	WBPH	Only water
3	50 µM GA + WBPH	WBPH	50 µM GA
4	100 µM GA + WBPH	WBPH	100 µM GA
5	50 µM MeJA + WBPH	WBPH	50 µM MeJA
6	100 µM MeJA + WBPH	WBPH	100 µM MeJA

**Table 3:** Primers and accession numbers of selected genes designed by NCBI for qRT-PCR.

S/No	Gene	Forward Primers	Reverse Primers	Accession No
1	*OsActin*	CTGCGGGTATCCATGAGACT	GGAGCAAGGCAGTGATCTTC	X16280.1
2	*OsRBBI3-3*	TCGTTCGTTCGATCATTCAG	TTTCCTCATGGTCCACACAA	AK243607
3	*OsPOX*	CCAGAATTTCAGGGACAGGA	TGCTGTAGTAGGCGTTGTCG	AK073202
4	*OsGAI*	CAGGTCATGTCCGAGGTGTA	CTCGCCTGTTTGTAGGCATT	AB030956
5	*OsGID2*	GGAGCGCAACCTTCTATCAG	AGACCTTGGACTCTGGAGCA	AK068248

## Data Availability

The data presented in this study are available on request from the corresponding author.

## Abbreviations

WBPH: white-backed planthopper; DAB, diaminobenzidine; GSH, glutathione; CAT, catalase; POD, peroxides; PPO, polyphenol oxidase; SA, salicylic acid; JA, jasmonate; ET, ethylene; GA, gibberellic acid; MeJA, methyl jasmonate; CK, cytokines; BR, brassinosteroids; ABA, abscisic acid; ROS, reactive oxygen species; PCR, polymerase chain reaction, HR, hypersensitive response; TAC, trichloroacetic acid; FW, fresh weight; TW, turgid weight; DW, dry weight; RWC, relative water content; EL, electrolyte leakage; MeOH, methanol.

## References

[1] Jan R., Khan M.A., Asaf S., Lee I.-J., Kim K.-M. (2020). Overexpression of OsF_3_H modulates WBPH stress by alteration of phenylpropanoid pathway at a transcriptomic and metabolomic level in *Oryza sativa*. Sci. Rep..

[2] Tang J., Cheng J., Norton G. (1994). HOPPER—An expert system for forecasting the risk of white-backed planthopper attack in the first crop season in China. Crop Prot..

[3] Reissig W. (1985). Illustrated Guide to Integrated Pest Management in Rice in Tropical Asia.

[4] Zhang H.-M., Yang J., Chen J.-P., Adams M.J. (2008). A black-streaked dwarf disease on rice in China is caused by a novel fijivirus. Arch. Virol..

[5] Pathak M.D., Khan Z.R. (1994). Insect Pests of Rice.

[6] Douglas A.E. (2006). Phloem-sap feeding by animals: Problems and solutions. J. Exp. Bot..

[7] Jan R., Khan M.A., Asaf S., Lubna, Lee I.-J., Kim K.-M. (2021). Over-Expression of Chorismate Mutase Enhances the Accumulation of Salicylic Acid, Lignin, and Antioxidants in Response to the White-Backed Planthopper in Rice Plants. Antioxidants.

[8] Verma V., Ravindran P., Kumar P.P. (2016). Plant hormone-mediated regulation of stress responses. BMC Plant Biol..

[9] Davière J.-M., Achard P. (2013). Gibberellin signaling in plants. Development.

[10] Macmillan J. (2001). Occurrence of Gibberellins in Vascular Plants, Fungi, and Bacteria. J. Plant Growth Regul..

[11] Staswick P.E., Tiryaki I. (2004). The Oxylipin Signal Jasmonic Acid Is Activated by an Enzyme That Conjugates It to Isoleucine in *Arabidopsis*. Plant Cell.

[12] Singh I., Shah K. (2014). Exogenous application of methyl jasmonate lowers the effect of cadmium-induced oxidative injury in rice seedlings. Phytochemistry.

[13] Jannoey P., Channei D., Kotcharerk J., Pongprasert W., Nomura M. (2017). Expression Analysis of Genes Related to Rice Resistance against Brown Planthopper, *Nilaparvata lugens*. Rice Sci..

[14] De Vleesschauwer D., Xu J., Höfte M. (2014). Making sense of hormone-mediated defense networking: From rice to *Arabidopsis*. Front. Plant Sci..

[15] Spoel S.H., Dong X. (2008). Making Sense of Hormone Crosstalk during Plant Immune Responses. Cell Host Microbe.

[16] Bari R., Jones J.D.G. (2009). Role of plant hormones in plant defence responses. Plant Mol. Biol..

[17] Nahar K., Kyndt T., Hause B., Höfte M., Gheysen G. (2013). Brassinosteroids Suppress Rice Defense against Root-Knot Nematodes Through Antagonism with the Jasmonate Pathway. Mol. Plant-Microbe Interact..

[18] Qin X., Liu J.H., Zhao W.S., Chen X.J., Guo Z.J., Peng Y.L. (2013). Gibberellin 20-Oxidase Gene *OsGA20ox3* Regulates Plant Stature and Disease Development in Rice. Mol. Plant-Microbe Interact..

[19] Yang D.-L., Li Q., Deng Y.-W., Lou Y.-G., Wang M.-Y., Zhou G.-X., Zhang Y.-Y., He Z.-H. (2008). Altered Disease Development in the *eui* Mutants and *Eui* Overexpressors Indicates that Gibberellins Negatively Regulate Rice Basal Disease Resistance. Mol. Plant.

[20] Ji H., Gheysen G., Denil S., Lindsey K., Topping J.F., Nahar K., Haegeman A., De Vos W.H., Trooskens G., Van Criekinge W. (2013). Transcriptional analysis through RNA sequencing of giant cells induced by *Meloidogyne graminicola* in rice roots. J. Exp. Bot..

[21] Ogawa M., Kusano T., Katsumi M., Sano H. (2000). Rice gibberellin-insensitive gene homolog, OsGAI, encodes a nuclear-localized protein capable of gene activation at transcriptional level. Gene.

[22] Zhang J., Luo T., Wang W., Cao T., Li R., Lou Y. (2017). Silencing *OsSLR1* enhances the resistance of rice to the brown planthopper *Nilaparvata lugens*. Plant Cell Environ..

[23] Gomi K., Sasaki A., Itoh H., Ueguchi-Tanaka M., Ashikari M., Kitano H., Matsuoka M. (2004). GID2, an F-box subunit of the SCF E3 complex, specifically interacts with phosphorylated SLR1 protein and regulates the gibberellin-dependent degradation of SLR1 in rice. Plant J..

[24] Zou X., Qin Z., Zhang C., Liu B., Liu J., Zhang C., Lin C., Li H., Zhao T. (2015). Over-expression of an S-domain receptor-like kinase extracellular domain improves panicle architecture and grain yield in rice. J. Exp. Bot..

[25] Agrawal G.K., Rakwal R., Jwa N.-S. (2002). Cloning and characterization of a jasmonate inducible rice (*Oryza sativa* L.) peroxidase gene, OsPOX, against global signaling molecules and certain inhibitors of kinase-signaling cascade(s). Plant Sci..

[26] Rakwal R., Agrawal G.K., Jwa N.-S. (2001). Characterization of a rice (*Oryza sativa* L.) Bowman–Birk proteinase inhibitor: Tightly light regulated induction in response to cut, jasmonic acid, ethylene and protein phosphatase 2A inhibitors. Gene.

[27] Laura B., Silvia P., Francesca F., Benedetta S., Carla C. (2018). Epigenetic control of defense genes following MeJA-induced priming in rice (*O. sativa*). J. Plant Physiol..

[28] Yang L., Han Y., Li P., Li F., Ali S., Hou M. (2017). Silicon amendment is involved in the induction of plant defense responses to a phloem feeder. Sci. Rep..

[29] Mittler R. (2002). Oxidative stress, antioxidants and stress tolerance. Trends Plant Sci..

[30] Corbineau F., Gay-Mathieu C., Vinel D., Côme D. (2002). Decrease in sunflower (*Helianthus annuus*) seed viability caused by high temperature as related to energy metabolism, membrane damage and lipid composition. Physiol. Plant..

[31] Wu G., Shortt B.J., Lawrence E.B., Leon J., Fitzsimmons K.C., Levine E.B., Raskin I., Shah D. (1997). Activation of Host Defense Mechanisms by Elevated Production of H_2_O_2_ in Transgenic Plants. Plant Physiol..

[32] Duan C., Yu J., Bai J., Zhu Z., Wang X. (2014). Induced defense responses in rice plants against small brown planthopper infestation. Crop J..

[33] Iftikhar A., Ali S., Yasmeen T., Arif M.S., Zubair M., Rizwan M., Alhaithloul H.A.S., Alayafi A.A., Soliman M.H. (2019). Effect of gibberellic acid on growth, photosynthesis and antioxidant defense system of wheat under zinc oxide nanoparticle stress. Environ. Pollut..

[34] Gangwar S., Singh V.P., Srivastava P.K., Maurya J.N. (2010). Modification of chromium (VI) phytotoxicity by exogenous gibberellic acid application in *Pisum sativum* (L.) seedlings. Acta Physiol. Plant..

[35] Wen F.-P., Zhang Z.-H., Bai T., Xu Q., Pan Y.-H. (2010). Proteomics reveals the effects of gibberellic acid (GA3) on salt-stressed rice (*Oryza sativa* L.) shoots. Plant Sci..

[36] Wang W., Zhou P., Mo X., Hu L., Jin N., Chen X., Yu Z., Meng J., Erb M., Shang Z. (2020). Induction of defense in cereals by 4-fluorophenoxyacetic acid suppresses insect pest populations and increases crop yields in the field. Proc. Natl. Acad. Sci. USA.

[37] Xu Y., Li K., Zhu K., Tian Y., Yu Q., Zhang W., Wang Z. (2020). Effect of exogenous plant hormones on agronomic and physiological performance of a leaf early-senescent rice mutant osled. Plant Growth Regul..

[38] Kim E.H., Kim Y.S., Park S.-H., Koo Y.J., Choi Y.D., Chung Y.-Y., Lee I.-J., Kim J.-K. (2009). Methyl Jasmonate Reduces Grain Yield by Mediating Stress Signals to Alter Spikelet Development in Rice. Plant Physiol..

[39] Anjum S.A., Tanveer M., Hussain S., Tung S.A., Samad R.A., Wang L., Khan I., Rehman N.U., Shah A.N., Shahzad B. (2015). Exogenously applied methyl jasmonate improves the drought tolerance in wheat imposed at early and late developmental stages. Acta Physiol. Plant..

[40] Siddiqui M., Khan M., Mohammad F., Khan M. (2008). Role of nitrogen and gibberellin (GA3) in the regulation of enzyme activities and in osmoprotectant accumulation in *Brassica juncea* L. under salt stress. J. Agron. Crop Sci..

[41] Gordy J.W., Leonard B.R., Blouin D., Davis J.A., Stout M.J. (2015). Comparative Effectiveness of Potential Elicitors of Plant Resistance against *Spodoptera frugiperda* (J. E. Smith) (Lepidoptera: Noctuidae) in Four Crop Plants. PLoS ONE.

[42] Qi J., Li J., Han X., Li R., Wu J., Yu H., Hu L., Xiao Y., Lu J., Lou Y. (2016). Jasmonic acid carboxyl methyltransferase regulates development and herbivory-induced defense response in rice. J. Integr. Plant Biol..

[43] Guan Y.R., Xue J.Q., Xue Y.Q., Yang R.W., Wang S.L., Zhang X. (2019). Effect of exogenous GA3 on flowering quality, endogenous hormones, and hormone-and flowering-associated gene expression in forcing-cultured tree peony (*Paeonia suffruticosa*). J. Integr. Agric..

[44] Yin Z., Chen J., Zeng L., Goh M., Leung H., Khush G.S., Wang G.-L. (2000). Characterizing Rice Lesion Mimic Mutants and Identifying a Mutant with Broad-Spectrum Resistance to Rice Blast and Bacterial Blight. Mol. Plant-Microbe Interact..

[45] Thordal-Christensen H., Zhang Z., Wei Y., Collinge D.B. (1997). Subcellular localization of H_2_O_2_ in plants. H_2_O_2_ accumulation in papillae and hypersensitive response during the barley—Powdery mildew interaction. Plant J..

[46] Asaf S., Khan A.L., Khan M.A., Imran Q.M., Yun B.-W., Lee I.-J. (2017). Osmoprotective functions conferred to soybean plants via inoculation with *Sphingomonas* sp. LK11 and exogenous trehalose. Microbiol. Res..

[47] Radhakrishnan R., Lee I.-J. (2013). Regulation of salicylic acid, jasmonic acid and fatty acids in cucumber (*Cucumis sativus* L.) by spermidine promotes plant growth against salt stress. Acta Physiol. Plant..

[48] Khan M.A., Ullah I., Waqas M., Hamayun M., Khan A.L., Asaf S., Kang S.-M., Kim K.-M., Jan R., Lee I.-J. (2019). Halo-tolerant rhizospheric *Arthrobacter woluwensis* AK1 mitigates salt stress and induces physio-hormonal changes and expression of *GmST1* and *GmLAX3* in soybean. Symbiosis.

[49] Sairam R.K., Rao K., Srivastava G. (2002). Differential response of wheat genotypes to long term salinity stress in relation to oxidative stress, antioxidant activity and osmolyte concentration. Plant Sci..

[50] Han Q.-H., Huang B., Ding C.-B., Zhang Z.-W., Chen Y.-E., Hu C., Zhou L.-J., Huang Y., Liao J.-Q., Yuan S. (2017). Effects of Melatonin on Anti-oxidative Systems and Photosystem II in Cold-Stressed Rice Seedlings. Front. Plant Sci..

[51] Jan R., Khan M.A., Asaf S., Lee I.-J., Bae J.-S., Kim K.-M. (2020). Overexpression of OsCM alleviates BLB stress via phytohormonal accumulation and transcriptional modulation of defense-related genes in *Oryza sativa*. Sci. Rep..

[52] Lee I.-J., Foster K.R., Morgan P.W. (1998). Photoperiod Control of Gibberellin Levels and Flowering in Sorghum. Plant Physiol..

